# PDE/ODE modeling and simulation to determine the role of diffusion in long-term and -range cellular signaling

**DOI:** 10.1186/s13628-015-0024-8

**Published:** 2015-10-14

**Authors:** Elfriede Friedmann

**Affiliations:** Department of Applied Mathematics, Im Neuenheimer Feld 294, Heidelberg, Germany

**Keywords:** Systems of coupled differential equations, Reaction-diffusion systems, Numerical simulation, Cellular signaling

## Abstract

**Background:**

We study the relevance of diffusion for the dynamics of signaling pathways. Mathematical modeling of cellular diffusion leads to a coupled system of differential equations with Robin boundary conditions which requires a substantial knowledge in mathematical theory. Using our new developed analytical and numerical techniques together with modern experiments, we analyze and quantify various types of diffusive effects in intra- and inter-cellular signaling. The complexity of these models necessitates suitable numerical methods to perform the simulations precisely and within an acceptable period of time.

**Methods:**

The numerical methods comprise a Galerkin finite element space discretization, an adaptive time stepping scheme and either an iterative operator splitting method or fully coupled multilevel algorithm as solver.

**Results:**

The simulation outcome allows us to analyze different biological aspects. On the scale of a single cell, we showed the high cytoplasmic concentration gradients in irregular geometries. We found an 11 % maximum relative total STAT5-concentration variation in a fibroblast and a 70 % maximum relative pSTAT5-concentration variation in a fibroblast with an irregular cell shape. For pSMAD2 the maximum relative variation was 18 % in a hepatocyte with a box shape and 70 % in an irregular geometry. This result can be also obtained in a cell with a box shape if the molecules diffuse slowly (with *D*=1 *μ*m^2^/*s* instead of *D*=15 *μ*m^2^/*s*). On a scale of cell system in the lymph node, our simulations showed an inhomogeneous IL-2 pattern with an amount over three orders of magnitude (10^−3^−1 pM) and high gradients in face of its fast diffusivity. We observed that 20 out of 125 cells were activated after 9 h and 33 in the steady state. Our in-silico experiments showed that the insertion of 31 regulatory T cells in our cell system can completely downregulate the signal.

**Conclusions:**

We quantify the concentration gradients evolving from the diffusion of the molecules in several signaling pathways. For intracellular signaling pathways with nuclear accumulation the size of cytoplasmic gradients does not indicate the change in gene expression which has to be analyzed separately in future. For intercellular signaling the high cytokine concentration gradients play an essential role in the regulation of the molecular mechanism of the immune response. Furthermore, our simulation results can give the information on which signaling pathway diffusion may play a role. We conclude that a PDE model has to be considered for cells with an irregular shape or for slow diffusing molecules. Also the high gradients inside a cell or in a cell system can play an essential role in the regulation of the molecular mechanisms.

## Background

A single cell, the smallest unit of life, alters, learns, adapts, specializes and differentiates. One of the causes of the diversification of a cell is the concentration distribution of signaling molecules which trigger signaling pathways. Changes in signaling pathways and in the amount of molecule concentration induce different reactions in the specific genes. These genes are controlled by proteins (transcription factors) and play an important role on the differentiation. They, in turn, control the concentration as well as the moment and duration of activation. To understand these biological processes it requires a detailed analysis of the signal transduction processes and their interplay. These complex processes are commonly described by mathematical models to generate experimentally testable hypotheses. With the available data different mathematical models can be derived. Initially in signal transduction, simple models consisting mainly of cascades of events were developed. Then, more general models followed to describe mechanisms such as pattern formation and regulation of immune response. A review on the state of the mathematical models in signal transduction can be found in [1]. In most cases, ordinary differential equations (ODE) are used to identify the role of specific components of the pathway and to determine the dynamics and outcome predictions. Partial differential equations (PDE) were used to analyze pattern formation for example stripe formation in zebras or fish [2], and cell structure properties like deformability, cell polarity [3] or cell migration [4].

In this paper, we use the reaction diffusion system for cellular signaling pathways to find the spatial distribution of the signaling molecules which causes the diversification of cells and their actions. Our contribution is to include the spatial aspect (size and geometry of the cell) of the data-based signal transduction models considered by our collaborators. Also we develop the mathematical models that describe the key dynamic properties and predict strategies for intervention. We extend a simple ODE-system to a more complicated PDE-system of reaction-diffusion equations. In PDE we take into account the traveling of the signal molecules from the outer membrane through the cytoplasm, thus forming a concentration gradient within the cell. The focus remains on the spatial distributions of the important signaling molecules whereas other processes can be replaced by specific measurements (e.g. the phosphorylated JAK (pJAK) concentration can be determined and replace the whole receptor model) or by ODE if we assume that the local domain (nucleus) of the interplay of the concentrations is considered to be well mixed (i.e. all processes take place everywhere).

The modeling leads to a coupled PDE/ODE system with Robin type boundary conditions. This non-standard model demands a substantial knowledge in mathematical theory. It needs the additional development of both analytical tools and numerical algorithms for its analysis, simulation and evaluation, which explains why the model is not commonly considered in other fields. Moreover, additional changes can occur on the dynamics of the pathway when a diffusion is included as shown in [5] for the Calvin-Cycle. The diffusion of the molecules can cause a gradient in their concentration which often plays an important role such as the one of RanGTP-Importin *β*. This gradient affects the stabilization of microtubules and the formation of the mitotic spindle [6, 7].

We are interested in biological processes regulated by diffusion of molecules over great distances (many cell distances) and a long period of time (over 200 min in the intracellular and 30 h in the intercellular pathways). We start with the classical diffusion model based on Fick’s law [8]. Given the diffusion coefficient the velocity of the diffusion depends on the nature of the medium, the size of the particles and temperature. In order to describe the complicated processes within a cell we consider a normal diffusion, an aided diffusion (such as energy-controlled movement or active transport with help of transport molecules) and a retarded diffusion (due to the binding process on other molecules). This leads to a nonuniform spatial distribution of molecules. Unfortunately, the current microscopy methods are not fully capable of measuring all dynamic parameters. Thus, diffusion maps inside the cell based on the experimental data are not yet available. In this paper we use the diffusion coefficient measured by our collaborators via Fluorescence Recovery After Photobleaching (FRAP) and Fluorescence Correlation Spectroscopy (FCS). In our models the diffusion coefficient has hence an extended physical meaning (it is a macroscopic approximation of the cytoplasmic processes) and can deviate from the one used in pure biomolecular simulations.

Our data-based modeling simplifies the system of equations because the certain biological phenomena can be replaced by a measured quantity (e.g. the receptor model in the JAK2/STAT5 pathway was skipped due to the measurements of the concentration of the phosphorylated JAK2-molecules) [9]. In the models we use the high-quality quantitative data from our collaborators [10]. This simplification is necessary for the theoretical treatment of the model and the numerics in three dimensions. The derived models have a structure allowing the mathematical theory to be suitable for several applications. For example, there is a particle exchange between two domains, one well-mixed domain (ODE model) and an adjacent non-well mixed domain (PDE model). Reactions occur in the well-mixed domain and reaction and diffusion occur in the non-well mixed domain.

In this paper we discuss whether the diffusion of signaling molecules plays a role in the signaling of intra- and intercellular systems. To illustrate this aspect we consider the models of the well-known pathways such as JAK2/STAT5, SMAD and IL-2. These non-standard models need the additional development on both analytical tools and numerical algorithms for further analysis, simulation and evaluation. The new analytical and numerical tools are presented in [11, 12]. The simulation results of the JAK/STAT pathway are presented for the first time in this work. We quantify the influence of the cell geometry and diffusivity on the dynamics of the signal. We also quantify the concentration gradients during the interplay between diffusion and degradation in order to discuss its effect to the cell fate. The biological interpretation of the simulation results are presented in a compact form to provide an overview in which pathways and cells the diffusion may play a role.

The paper has five main sections. After an introduction to the subject in the first section, the section Methods presents two of our mathematical tools: modeling and numerical simulations. We present the general mathematical models which differ from the commonly used models for these applications. We will give a short overview of their mathematical analysis and numerical calculations to underline the importance of the theoretical background. We present our numerical methods based on finite elements which are used to simulate the modeled equations. We use a different method to solve the stronger nonlinear coupling in the inter-cellular model. Therefore, each of the section Results and Discussion comprises two subsections where we examine models with intra- and inter-cellular diffusion separately. An analysis of the numerical methods used will be subject of a forthcoming paper. The section Discussion contains the observations and the biological interpretation of the numerical results.

## Methods

### Model setup

Since the process inside a cell is highly complex all the existing models are only an attempt of an approximation. Membrane receptors receive extracellular signals from each cell in form of protein concentrations. The signals are processed, encoded and transferred to the nucleus where a further development is decided. This signal processing from the cell membrane to the nucleus occurs via activation (phosphorylation) and spatial relocation of the components of the signaling pathways. The cytoplasmic proteins are recruited to the cell membrane where the activation takes place. The activated molecules dissociate from the receptor, dimerize, trimerize or form other complexes and move to the nucleus to regulate the transcription of target genes. Then, the molecules are deactivated and are shuttled back to the cytoplasm where the whole process restarts until the cell cycle in the considered pathway is regulated. The three factors that influence the process are the fast diffusion rate, the activation rate and the boundary conditions at the interface between cytoplasm and nucleus which determine a nuclear accumulation of the activated molecules. A steady state is reached by the continuous molecule shuttling.

In [6] it shows that the spatial separation of activation and deactivation mechanisms can result in steep phospho-protein gradients. For spherical cells, a single concentration and cytosolic phosphatase, diffusion must be taken into account if *D*/*L*^2^<<*k*, where *L* describes the traveled distance and *k* the dissociation rate of the molecules. In [12] it shows that this estimation can be used for our model in the case of cells with a regular shape. For general cases and non-linear models this estimation can not be applied, and we need three dimensional simulations for each considered pathway. The cell fate is not only determined by the cell itself but also by surrounding cells (cell-to-cell communication) and molecules and a complex interplay between them. We will present the models for signaling pathways inside a cell (intra-cellular signaling pathways) and between cells (inter-cellular signaling pathways) that appear to have the same mathematical structure.

#### The coupled PDE/ODE reaction diffusion model

Let *x*∈*Ω* and *t*∈ [0,*T*], 0≤*T*<*∞* be two independent variables (spatial and temporal respectively) and *u*_*i*_(*t*,*x*) denote the spatial-temporal molecule concentrations (*i*=0,1,…,*n*) involved in the specific pathway at position *x* and time *t*, $n\in \mathbb {N}$. Also let $\,\Omega \subset {\mathbb {R}}^{3}\,$ be the space resolved part (e.g. cytoplasm or extracellular space) and thus bounded by a sufficiently smooth boundary *∂Ω*. We use the standard notation of space-time function spaces. For a real function Banach space *X* with norm ∥·∥ on *Ω*, the space *L*^*p*^(0,*T*;*X*) consists of all measurable functions *u*:[0,*T*]→*X* with

(1) $$ \|u\|_{L^{p}(0,T;X)}:= \left({\int_{0}^{T}} \|u(t)\|^{p}\,dt\right)^{1/p} < \infty,  $$

for 1≤*p*<*∞*, and $\,\|u\|_{L^{\infty }(0,T;X)} := \text {ess sup}_{(0,T)} \,\|u(t)\|<\infty $. We denote by (·,·)_*Ω*_ and (·,·)_*Γ*_ the usual *L*^2^ scalar products over the domain *Ω* and a part *Γ*⊂*∂Ω* of its boundary, respectively, and by ∥·∥_*Ω*_ and ∥·∥_*Γ*_ the corresponding norms.

The general structure of our models looks like a coupled two-compartment model. In *Ω* we have reaction-diffusion equations (PDE) for each diffusing concentration *u*_*i*_ with diffusion rate *D*_*i*_≥0, *i*=0,…,*m* :

(2) $$ \begin{aligned} \partial_{t} u_{i}&= D_{i} \Delta u_{i} -f_{i}(u_{i},u_{j})\quad \mathrm{in }\quad\Omega\times(0,T],\\ \quad j&=m+1,\ldots,n. \end{aligned}  $$

The functional *f* describes the kinetical part of the law of mass action and the coupling with involved concentrations *u*_*j*_ entering the domain *Ω*. We potentially have a nonlinear Robin boundary condition on a partial boundary *Γ*⊂*∂Ω* :

(3) $$ D_{i} \partial_{n} u_{i}= g_{i}(u_{i},u_{j}) \quad\mathrm{on }\quad\Gamma\times(0,T].  $$

For the mathematical correct formulation we impose an initial condition from measurement:

(4) $$ u_{i}(0,x)= {u_{i}^{0}} \quad\mathrm{in }\quad\Omega.  $$

Under the assumption of the well-mixed molecule concentrations over a certain domain (for example in the nucleus in our intracellular signaling models or in the entire cell in our intercellular signaling models), we have ODE:

(5) $$ \partial_{t} u_{j}= h_{j}(\bar{u}_{i},u_{j})\quad \mathrm{in }\quad(0,T],\quad j=m+1, \ldots,n,  $$

with the space-independent averaged value $\bar {u}_{i}=\frac {1}{|\partial \Omega |}\int _{\partial \Omega } u_{i}(t,s)ds$ and initial conditions

(6) $$ u_{j}(0)= {u_{j}^{0}}.  $$

The detailed model description for the JAK2/STAT5 signaling pathway can be found in [9], the SMAD pathway in [12] and the IL-2 signaling in [13–16].

The mathematical analysis and numerical simulation of such systems can not be performed with a standard theory. The methods depend very much on the variety factors such as structure of the equations, nonlinearities, coupling, domain and more. For a linear model for JAK2/STAT5, we showed the well-posedness and the properties of the solution for reliable numerical simulation [11]. In [17] analytical investigations are performed for a simplified situation of two cells in two dimensions. In [14, 17] numerical simulations were performed in two dimension by using Finite Differences (FD). Nevertheless, the two dimensional results represent an artificial reality, e.g. the 2D cells are infinitely-long cylinders instead of spheres. The cytokine Interleukin 2 (IL-2) can spread only in two directions causing the change in its amount and gradient. For a better understanding of the process, realistic simulations in three dimension are necessary [13].

In the next section we present the numerical simulations of (2)–(6).

### Numerical simulations

#### General description of the methods

We implement the coupled PDE/ODE system in the Finite-Element-platforms developed in our group (Gascoigne [18] and deal.II [19]). The principal components of our numerical methods are:

- Galerkin space discretization by Finite Elements (FE)
- segregating solution approach [12] or a fully coupled multilevel algorithm as solver [13]
- multilevel preconditioner with a Block-Gauss-Seidel scheme as smoother consisting of damped Jacobi iterations on the PDE part (presented in a forthcoming paper)
- error control techniques with the Dual Weighted Residual (DWR) method for space and time adaptive discretization [20, 21].

A segregating solution approach is a splitting solution approach. It is often used when the restriction on accuracy can be relaxed in order to allow an easier numerical treatment of complicated problems. Such an approach makes it possible to reuse the existing solvers and is widely used in numerical methods for coupled systems. The equations are discretized via the Rothe Method, first on temporal discretization by applying backward Euler or a similar solver of second order, then on spatial discretization via Finite Elements. The two parts are solved sequentially with splitted operators which keep the storage space low. In case of a strong coupling, it requires a very small time step which leads to a long computing time. Therefore, we developed an adaptive and fully coupled solver for the inter-cellular model.

Because the main focus of this paper is devoted to biological diffusion, we will keep the mathematical aspects short. Interested readers are recommended to look trough the literature cited.

## Results

### Intra-cellular diffusion

**Fig. 1:**
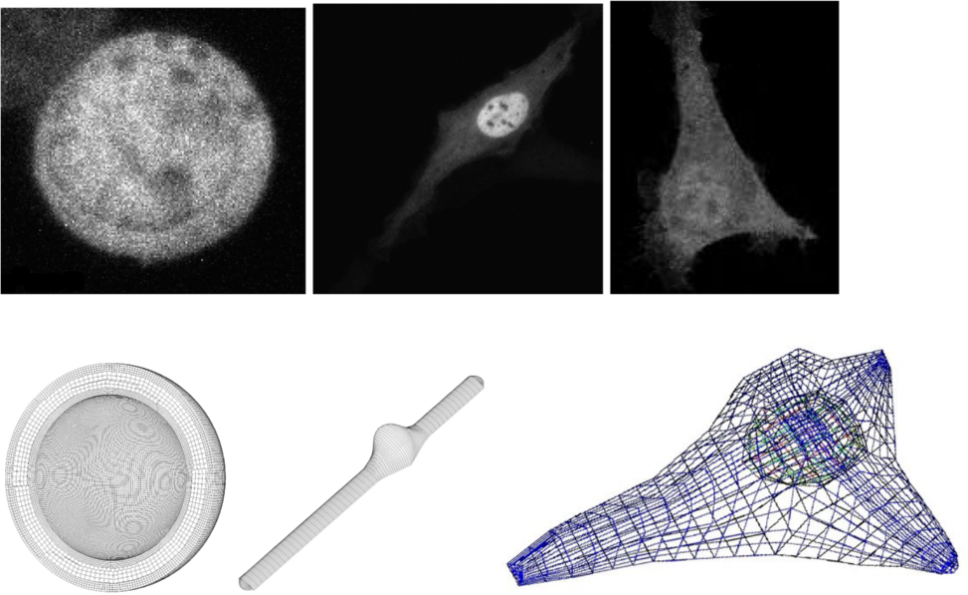
Considered geometries of the cells. *Left*: the sphere-type CFU-E, *middle*: the modeled fibroblast, *right*: the reconstructed fibroblast from microscopy data

**Fig. 2:**
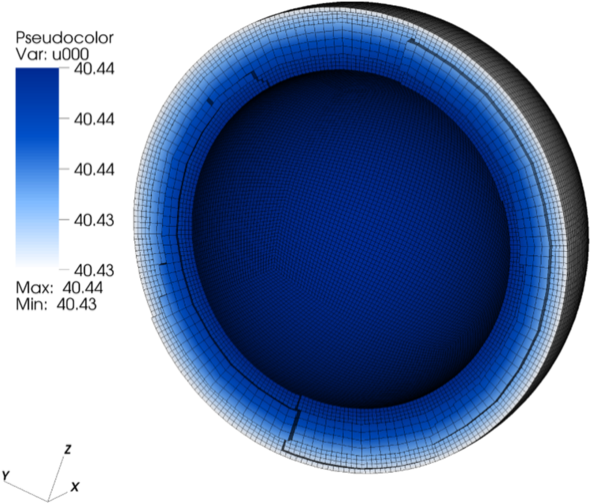
Concentration distribution in the CFU-E cell. Homogenious STAT5-concentration distribution. There is no deviation with respect to the pure ODE model; everything is well-mixed
- a mathematical model of fibroblast (NIH3T3, Figs. 1, 3 and 6)

Our model of the JAK2/STAT5 signaling pathway and the parameters can be found in [9]. The STAT5 molecules form dimers in both activated and non-activated states. If our concentration variables describe the dimers instead of the monomers, the resulting model equations are linear with a unique solution [11]. We consider the model for different cell types and different cell geometries:

- a sphere-type primary cell (CFU-E, Figs. 1 and 2)

**Fig. 3:**
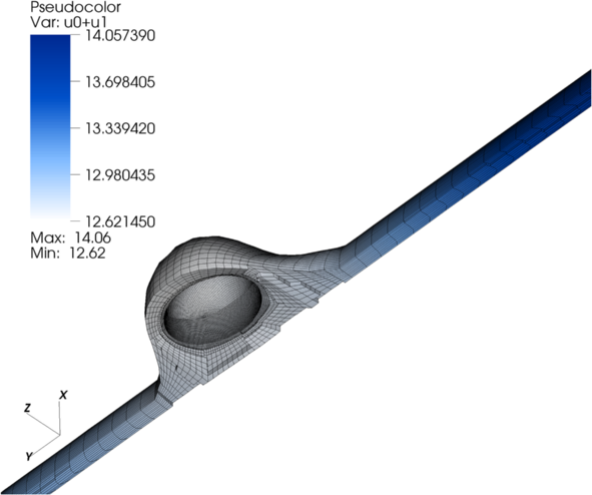
Concentration distribution in the modeled fibroblast. Nonhomogenious total STAT5-concentration distribution: 11 % gradient in the total STAT5-concentration. Deviation to the ODE model: 0.7 molecules/*μ*m^3^
- a reconstructed fibroblast via microscopy data (Figs. 1 and 4)

**Fig. 4:**
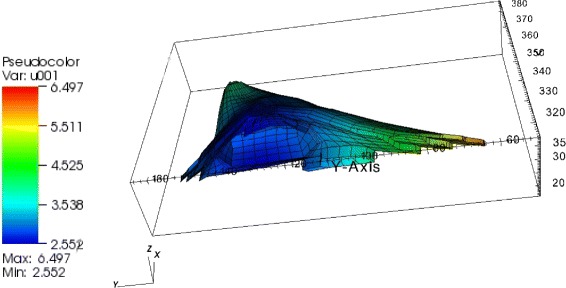
Concentration distribution in the reconstructed fibroblast. Nonhomogeneous pSTAT5-concentration distributin: 10 % gradient in the phosphorylated STAT-concentration. Deviation to the ODE model: 0.07 molecules/*μ*m^3^. The figure shows a cut in the (y,z)-plaine, only half of the fibroblast
- an artificial cell where we added two cytoplasmic extensions (filopodia) to the reconstructed geometry (Fig. 5).

**Fig. 5:**
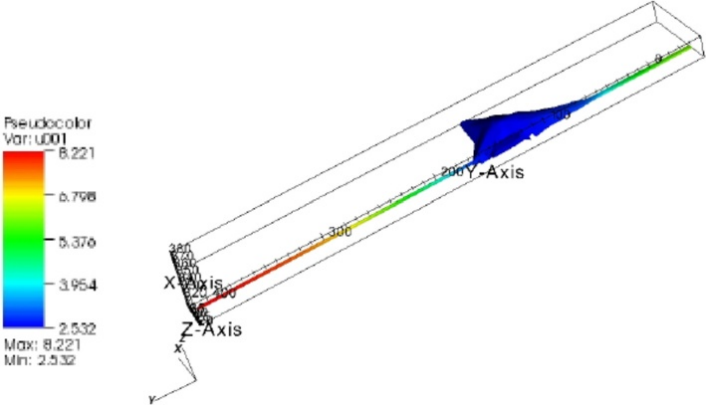
Concentration distribution in the artificial fibroblast with extensions. Nonhomogenious pSTAT5-concentration distribution: 70 % gradient in the total STAT-concentration. Deviation to the ODE model: 0.35 molecules/*μ*m^3^

The model for the different cell types requires different input parameters. Some parameters were measured by specific experimental methods and others were estimated [9]. Fibroblasts can have various shapes often depending on the current function or tissue concentration [12]. This has to be considered in modeling as well as in the experiments when a certain parameter is measured.

To examine the spatial distribution of the diffusive molecules, activated and non-activated STAT5 in the cytoplasm (*u*_0_,*u*_1_), we evaluate the maximum relative variation of a concentration at a given time point t:

(7) $$ \max\limits_{x\in\Omega} \text{var}(u_{i}) = \frac{\max\limits_{x\in\Omega}u_{i}(x) - \min\limits_{x\in\Omega}u_{i}(x)}{\max\limits_{x\in\Omega}u_{i}(x)}, \quad i=0,1.  $$

One of the advantages of numerical simulations (in silico experiments) is that we gain additional information about the processes modeled. The outcome of the simulation can be adapted to meet the requirement of the experiment. For this pathway both states of the molecules (non- and activated state, *u*_0_+*u*_1_) must be marked and measured together. The quantity of each specie or other quantities of interest that can not be evaluated in the experiment can be determined from the simulation.

**Fig. 6:**
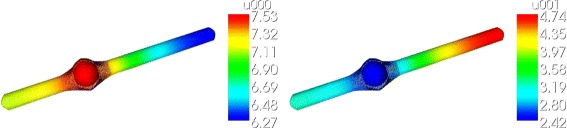
Anisotropic diffusion. In case of anisotropic diffusion we observe also a nonhomogenious concentration distribution: 11 % gradient in the total STAT-concentration and a greater deviation to the ODE model: 0.42 molecules/*μ*m^3^ (**×** **6**) at t = 10 min and 0.23 molecules/*μ*m^3^ (**×** **3**) in the steady state

The results of the simulation of the JAK2/STAT5 model are presented in the Figs. 2, 3, 4, 5 and 6.

#### Result 1.

For a perfectly spherical cell such as the CFU-E cell with a large nucleus compared to the amount of cytoplasm, we observed a homogeneous distribution of the signaling molecules due to the fast diffusion (Fig. 2). For this geometry we observed no deviation between the ODE and the PDE model.

#### Result 2.

For the modeled fibroblast we observed a maximum relative variation of 11 *%* of the total STAT-concentration. The concentration in the cytoplasm was about 1.5 molecules/*μ*m^3^ higher compared to the outcome of the pure ODE model due to the diffusion of the molecules into the nucleus. The activation process takes a place at the cell receptors located at the outer membrane which concludes that the main contribution to this concentration variation comes from the activated STAT5-concentration. Comparing the results of the ODE model with the PDE model the largest difference in the activated STAT5-concentration was 0.7 molecules/*μ*m^3^ for the modeled fibroblast (Fig. 3). This amount occurred 10 min after starting the activation process. During 200 min of simulation a steady state was observed in the last 80 min.

#### Result 3.

The simulation result for the reconstructed geometry lies in between the results of the CFU-E cell and the modeled fibroblast. Here, we obtained a maximum relative variation of 10 *%* of the activated STAT-concentration, a variation of 2−3.5 *%* of the non-activated STAT-concentration and a concentration difference of 0.07 molecules/*μ*m^3^ between the ODE and PDE model (Fig. 4).

#### Result 4.

The greatest relative concentration variation was observed in the artificial cell assembled with the two extensions: 40 *%* for the unphosphorylated STAT5-molecules and 70 *%* for the phosphorylated (Fig. 5). The deviation in the pSTAT5-concentration between the two models (ODE-PDE) was five times greater (0.35 molecules/*μ*m^3^) than in the same cell without extensions.

A cell has a complex structure thus the diffusion of the molecules therein is not isotropic like in homogeneous materials, i.e. the same diffusion in every direction. In some parts of the cytoplasm there is a retardation in the motion of molecules possibly caused by binding processes and a speed-up by active transport or aided diffusion. Especially in signaling pathways, the molecules are recruited towards the nucleus. Often they are transported by motor-proteins along the microtubules, so that the diffusion can be faster in direction to the nucleus. To model this biological behavior we consider the convection-diffusion equation with a time and space dependent diffusion tensor $\tilde {D}(t,x)$. For *i*=0,…,*m* and *j*=*m*+1,…,*n*, we have

(8) $$ \begin{aligned} \frac{\partial u_{i}}{\partial t} &= \nabla \cdot (\tilde{D}_{i}(t,x) \nabla u_{i}) - \nabla \cdot ({v} u_{i})\\&\quad-f_{i}(u_{i},u_{j}), \quad \mathrm{in }\quad\Omega\times(0,T]\!. \end{aligned}  $$

*v* is the velocity of the motor-proteins. To determine *v* it requires additional experiments. As a first approximation, we use the anisotropic diffusion equations with an inhomogeneous but constant diffusion tensor for each diffusing species:

(9) $$ \frac{\partial u_{i}}{\partial t}= \nabla\cdot(\tilde{D}(t,x)\nabla u_{i}) -f_{i}(u_{i},u_{j})  $$

with

$$\tilde{D}(x,t)=\left(\begin{array}{ccc} \tilde{D}_{xx} & 0 & 0\\ 0 & \tilde{D}_{yy} & 0\\ 0 & 0 & \tilde{D}_{zz} \end{array}\right), \ x\in\Omega. $$

For the isotropic diffusion in Eq. (2) we used the same diffusion coefficient for the activated and non-activated STAT5, $D_{i}=\bar {D}$, *i*=0,1.

$$\bar{D}=\left(\begin{array}{ccc} D & 0 & 0\\ 0 & D & 0\\ 0 & 0 & D \end{array}\right). $$

#### Remark.

We choose the coefficients $\tilde {D}_{\textit {xx}}, \tilde {D}_{\textit {yy}}$ and $\tilde {D}_{\textit {zz}}$ so that we have higher diffusion towards the nucleus $\tilde {D}_{\textit {yy}}>> D$ and smaller diffusion elsewhere $\tilde {D}_{\textit {xx}}= \tilde {D}_{\textit {zz}}<<D$. Also we want the same trace in the diffusion tensors, i.e. $\tilde {D}_{\textit {xx}}+\tilde {D}_{\textit {yy}}+\tilde {D}_{\textit {zz}}=3D$. The results are presented in Fig. 6:

#### Result 5.

For spatial inhomogeneous diffusion of the molecules we obtained the same maximum relative variation of the total STAT-concentration (1.5 molecules/*μ*m^3^). However, the deviation between the ODE and PDE model was six times greater after 10 min of activation (0.42 molecules/*μ*m^3^) and three times greater in the steady state (0.23 molecules/*μ*m^3^) than in the cell with isotropic diffusion. This coincides with the fact that some molecules reach the nucleus faster but others have a longer sojourn time in the cytoplasm.

**Fig. 7:**
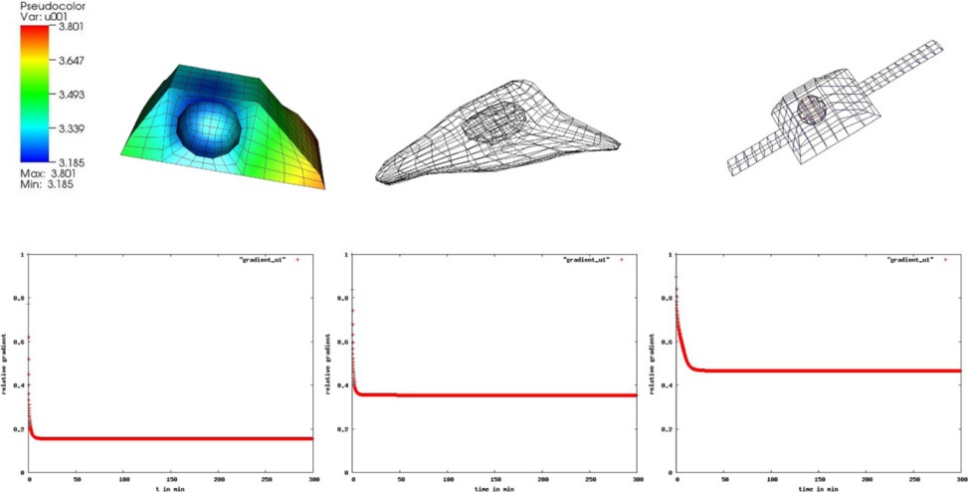
Gradients in the pSMAD2-concentration. We observe a 18 % gradient in the pSMAD2 concentration in the box shaped cell, a 38 % gradient in the reconstructed and a 50 % gradient in the artificial cell with extensions
- a reconstructed geometry from microscopy data (Fig. 7 central)
- an artificial geometry assembled with two cytoplasmic extensions (Fig. 7 right).

As a second intra-cellular signaling pathways we analyze the SMAD signaling pathway in hepatocytes. Details of the model and the biological function of the pathway can be found in [12]. The SMAD-molecules undergo a more complex oligomerization. They form dimers and trimers and react with other molecules building multi-complexes that are described by non-linear and more complex equations. For the cell shape we also consider various geometries:

- a mathematical model of hepatocyte (Fig. 7 left)

The hepatocyte model represents a cell from the liver. It has a regular box-shape due to the high cell density in the tissue. The reconstructed cell is flatter and elongated due to microscopy reconstruction from a cell culture with a less-dense cell concentration [12]. In the artificial cell we added two cytoplasmic extensions to the modeled geometry to see any possible effect of these cytoplasmic structures which commonly appear in less-dense cell cultures.

#### Result 6.

We observed a much greater maximum relative concentration variation in the activated SMAD concentration than in the activated STAT concentration in the JAK2/STAT5 pathway. For the activated SMAD2 we obtained 18 % in the box shaped cell, 38 % in the reconstructed and 50 % in the artificial cell [12].

**Fig. 8:**
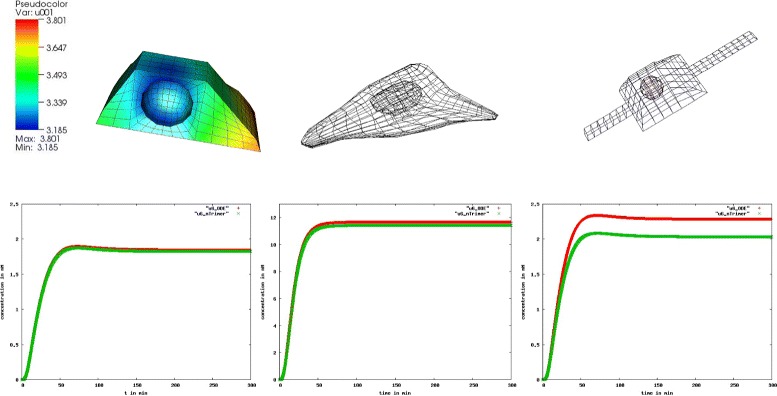
Deviation in the nuclear trimer concentration. We observe no deviation in the nuclear trimer concentration in the box shaped cell, a negligible deviation in the reconstructed and a clear visible deviation in the artificial cell with extensions: ODE (red) and PDE (green)

To observe the influence of the cytosolic gradients on any further processes concerning gene regulation we analyze the difference in the nuclear trimer concentration which binds to the DNA. Figure 8 shows the result as follows:

#### Result 7.

A visible difference in the nuclear SMAD-trimer concentration between the ODE and PDE model was seen only in the artificial cell with extensions.

**Fig. 9:**
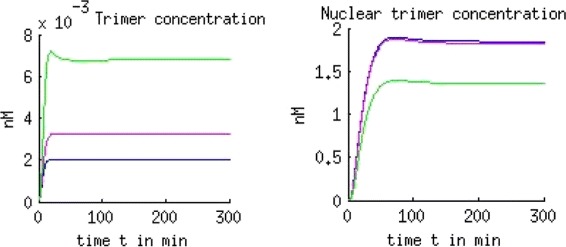
Deviation in the cytoplasmic and nuclear trimer concentration. The deviation in the cytoplasmic concentration of the trimer is compensated by a deviation in the nuclear trimer concentration: ODE (blue), PDE with *D*=15 *μ* *m* ^2^/*s* (pink) and PDE with *D*=1 *μ* *m* ^2^/*s* (green). The nuclear trimer concentration is of three orders of magnitude higher than the cytoplasmic trimer concentration (nuclear accumulation)

For the further investigation on this particular case where the greater gradients in the cytoplasm have only small effect on the amount of concentration in the nucleus we compare the total amount of SMAD-trimers in the two compartments (Fig. 9):

#### Result 8.

For the modeled hepatocyte with a regular form we observed no visible difference in the nuclear SMAD-trimer concentration between the ODE and PDE model except for slower diffusing molecules.

Different diffusion coefficients give rise to different concentration distributions:

#### Result 9.

**Fig. 10:**
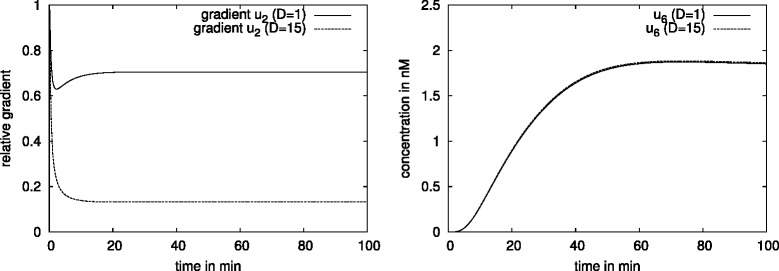
Gradient in the cytosolic SMAD-trimer and amount of the nuclear trimer concentration for the regular formed hepatocyte. We observe a 70 % gradient (left) of cytosolic SMAD-trimer in the regular formed hepatocyte using *D*=1 *μ* *m* ^2^/*s* (solid line) and a 18 *%* gradient using faster diffusion, *D*=15 *μ* *m* ^2^/*s* (dotted line). A difference in the diffusion coefficient of one order of magnitude does not influence the nuclear trimer concentration, and the huge gradient in the cytoplasm seems having no influence in the nucleus which is an indication of nuclear accumulation

A slower diffusion of the cytosolic trimer with *D*=1 *μm*^2^/*s* instead of *D*=15 *μm*^2^/*s* implied a 70 % cytosolic SMAD-trimer concentration variation in the steady state (Fig. 10) compared to 18 % as seen in the previous result.

We will give a more detailed discussion about the size of cytosolic gradients and their effect to the development of the cell in the next section, Discussion about biological diffusion.

### Inter-cellular diffusion

Our model captures the first 30 h of IL-2 signaling, the initial phase after antigen stimulation where the cells are primed for proliferation but have not yet entered initiated cell division. Biological details can be found in [14–16].

**Fig. 11:**
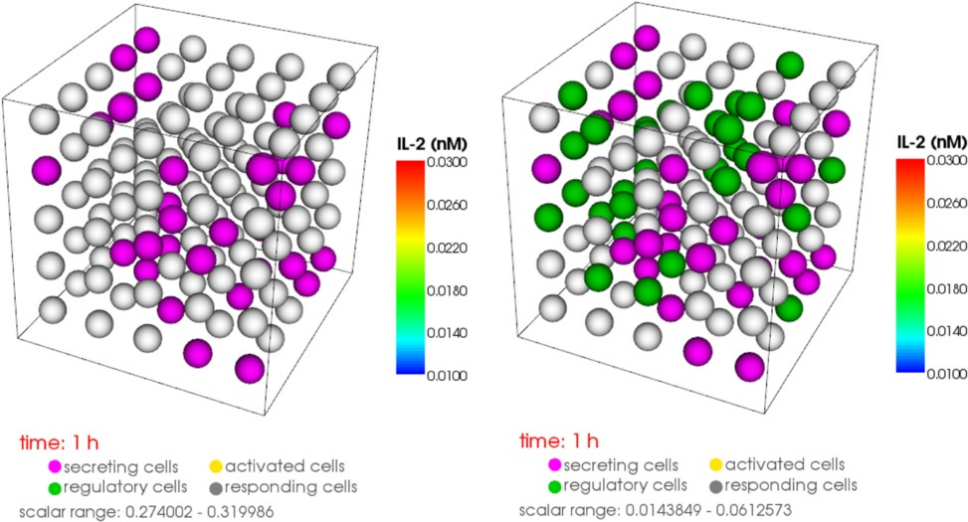
Schemes of the calculation domain. Prototype of our calculation domain which represents a cubic cutout of the 3D-lymph node containg 125 T-cells from which 25 % are secreting cells (*left, right*) and 25 % regulatory cells (*right*)

For numerical calculations we choose a part of the three dimensional lymph node with 125 or 218 cells (Fig. 11). 25 % of these cells are randomly chosen as secreting IL-2 (secretory T cells) and the rest of the cells are absorbing IL-2 (T helper and regulatory cells). The cells compete for IL-2 and those who absorb more will upregulate their receptors and consume even more IL-2. So called regulatory T cells have a higher absorbing rate and may downregulate the signal, for example to avoid autoimmune reactions. The interaction of the cells generates a space and time dependent dynamics which describes the competition of the cells for IL-2. This dynamics depends on the position of the different cell types and stabilizes after 30 h in an inhomogeneous steady state. The winner cells are activated as a large number of receptors (more than 4000 receptors after 30 h of simulation time) are formed on the surface.

#### Result 10.

**Fig. 12:**
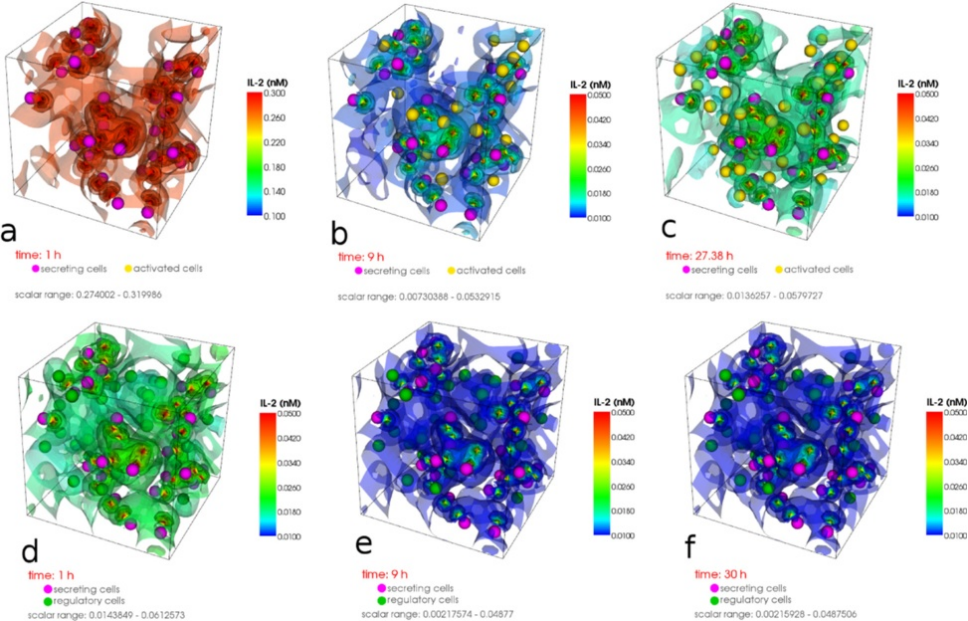
Spatial distribution of IL-2 in the extracellular domain. **a** 31 (of 125) secreting cells, high IL-2 distribution all over at *t*=1*h*, **b** lowest mean IL-2 distribution, heterogenous pattern, 20 activated cells at *t*=8*h*, **c** heterogenous IL-2 distribution, 33 activated cells in the steady state at *t*=30*h* **d** 31 secreting cells, 31 regulatory T-cells (of 125), low IL-2 distribution after *t*=1*h*, **e** very low IL-2 concentration after *t*=8*h*, no cell activated, **f** very low IL-2 concentration after *t*=30*h*, no cell activated. The IL-2 distribution is visualized by isosurfaces obtained from topological methods [22]

In Fig. 12 the particular isosurfaces of the IL-2 distribution are presented in the extracellular space at specific time points (after 1, 9 and 30 h of activation) obtained by topological methods [22]. We confirmed the fast-diffusivity of IL-2; after only an hour IL-2 diffused everywhere in a high concentration. Then the local chemical reactions on each cell start to determine the behavior of the dynamics. If a cell has a greater chance to absorb more IL-2 it express more receptors. Due to the higher amount of expressed receptors the cell absorbs even more IL-2 so that in its vicinity there is not enough IL-2 left for the other cells to be activated. Consequently, an inhomogeneous IL-2 pattern and high concentration gradients are formed. In our model we have 31 secretory cells out of 125. After 9 h of activation we observed 20 activated cells and 33 cells after 30 h in the steady state. The same simulations with 31 regulatory T cells showed an IL-2 concentration of one order of magnitude (ten times) lower after one hour (comparison Fig. 12a) with d)). After 9 h we observed a very low IL-2 concentration and no activation. This behavior remains until the steady state. We can conclude that in our model 31 regulatory T cells can completely downregulate the signal.

#### Result 11.

**Fig. 13:**
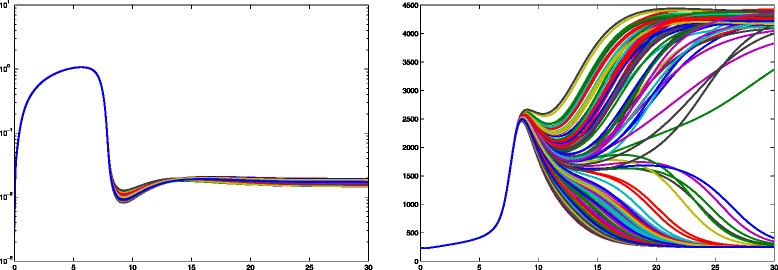
Distribution of IL-2 and receptors on the cell surfaces. *Left*: amount of IL-2 in pM on the cell surfaces, *right*: IL-2R expression level of non-secretory Th cells. 60 cells (from 218) are above 4000 IL-2Rs and thus considered as activated

The Fig. 13 left shows the IL-2 distribution on the surface of 218 cells. The concentration varies over three orders of magnitude. The Fig. 13 right shows the expression level of the cells: 60 out of 218 cells have more than 4000 IL-2 receptors and are thus considered as activated. By introducing 54 regulatory cells into the lymph node this activation would be suppressed completely.

**Fig. 14:**
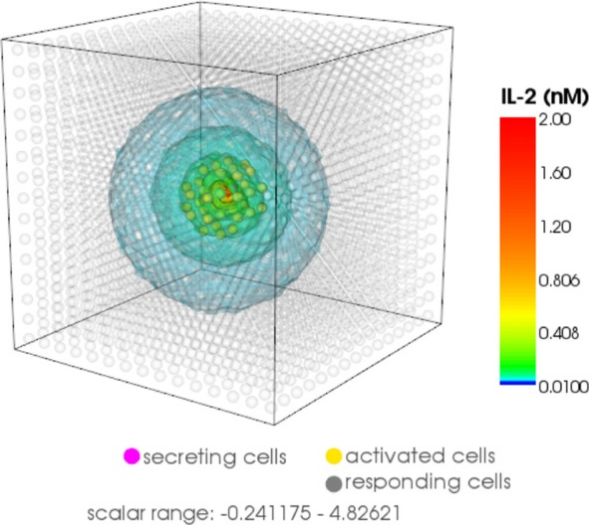
IL-2 range. Only 37 cells out of 3374 (marked gold) are activated from a single secreting one located in the middle. We used *q*=10^6^ molecules/h. The diffusion of IL-2 is fast, it spreads inside the domain such that a few cells can be activated

To analyze the range of action of the cytokine IL-2 we constructed a three dimensional in silico experiment containing 3375 cells (Fig. 14). The cell in the middle is the single secreting cell in the system and all others are naive T helper cells which can be activated when enough IL-2 is available.

#### Result 12.

The Fig. 14 shows that only 37 cells were activated in immediate vicinity of the secreting cell (marked with yellow) by a secretion rate of 10^6^ molecules/h which confirms the strong localized uptake of IL-2. Our calculations showed that with a higher secretion rate (1.5×10^6^ molecules/h) 123 cells were activated.

In the next section we will discuss the importance of the modeled biological diffusion for the intra- and as well for the inter-cellular signaling pathways.

## Discussion about biological diffusion

### Intra-cellular diffusion

Our simulations give us the potential to analyze the effect of diffusion of signaling molecules. The signal to the nucleus may be delayed or modulated by diffusive processes which causes changes in gene expression. The possibility of an impact of the changed signal on the further development of the cell has to be analyzed separately in each model.

In case of the SMAD signaling pathway we investigate further on the role of diffusion. In addition to the nuclear accumulation, our intra-cellular models exhibit mass conservation. The molecules change the compartments (cytoplasm and nucleus), their state (non-activated to activated and vice versa) and interact with each other while their number is preserved. The deviation of the concentrations (pSMAD2, trimer) in the cytoplasm is balanced by a deviation of the corresponding concentrations in the nucleus. Due to the nuclear accumulation any remarkable cytoplasmic concentration deviation is slightly small in the nucleus for cells with regular shape. In this case enough transcription factors may be available in the nucleus and do not have to compete for the binding sides of the DNA except for the case where the molecules diffuses slower than *D*=1 *μm*^2^/*s*. For a diffusion coefficient *D*=15 *μm*^2^/*s* we observed in Fig. 9 no change in the nuclear concentration in the PDE model compared to the ODE model and for *D*=1 *μm*^2^/*s* a small change with a possible effect to the signal which we will discuss later.

#### Result 13.

Our simulation results showed that in signaling pathways with nuclear accumulation the size of cytoplasmic gradients does not indicate the change in gene expression. For cells with regular shape the effect of cell geometry to the signal can be neglected. The concentration difference in the nucleus where gene expression occurs is small and the effect to the further development of the cell is not known yet.

**Fig. 15:**
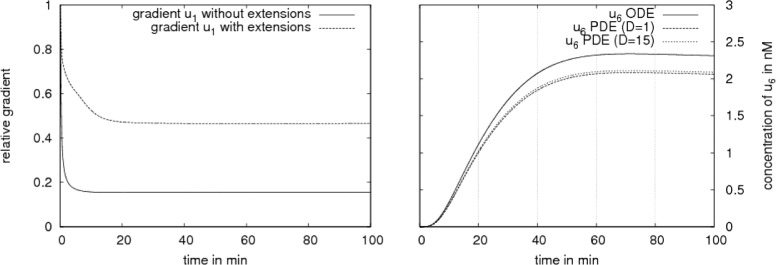
Gradient in the cytosolic pSMAD2 and amount of the nuclear trimer concentration for the hepatocyte with extension. We observe a 16 % gradient (left) of cytosolic pSMAD2 in the modeled hepatocyte (solid line) and a 45 *%* gradient in the cell with extensions (dotted line). The amount of nuclear trimer concentration (right) in the steady state is lower for the cell with extensions, the molecules sojourn longer in the cytoplasm until they reach the nucleus

Figure 10 demonstrates that different sizes of gradients in the cytoplasm (70 %, 18 % respectively) caused by two different diffusion coefficients (*D*=1 *μm*^2^/*s*, *D*=15 *μm*^2^/*s*) give rise to the same dynamics in the nuclear trimer concentration. For the considered set of parameters our numerical simulations show that the diffusion of molecules in the cytoplasm always plays a role in cells with an irregular shape like dendritic cells or cells with cytoplasmic extensions. In case the diffusion coefficient *D* is of a few orders of magnitude lower than in the considered pathways (*D*<<15 *μm*^2^/*s*), visible effects may appear in the dynamics also for cells with regular shape. The deceleration of diffusion of a factor 10 does not have an effect (Figs. 10 and 15). This gives an idea that for our applications the measurement of diffusion coefficients allows more flexibility.

#### Result 14.

In a cell with a regular shape the slow diffusing molecules (*D*<1 *μm*^2^/*s*) cause the change in the nuclear concentration.

**Fig. 16:**
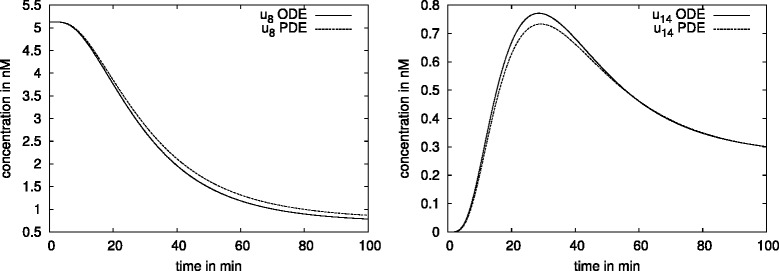
Deviation of the nuclear SnoN- and SnoN-SMAD-tetramer-concentration in the hepatocyte with extensions. Comparison of ODE (solid line) and PDE (dotted line) for the nuclear SnoN (*left*) and SnoN-SMAD tetramer protein complex (*right*) in the artificial hepatocyte with extensions

Figure 15 shows that the cell with extensions exhibits a greater relative concentration variation in the cytoplasmic concentration (45 %) and also a visible deviation in the nuclear trimer concentration. The molecules are activated on the outer membrane and need a longer time to travel through the cytoplasmic dendrite to the nucleus. This implies that less molecules are available for the processes in the nucleus, and diffusion can change gene expression in a cell with irregular shape. This is explained by the inhibition of pSMAD2 and trimer nuclear accumulation in [12]. SMAD2 molecules persist longer in the cytoplasmic domain. Therefore, the chance for other proteins to compete for SMAD2 binding increases. In the end less molecules will be available in the nucleus to bind to the DNA. Nevertheless, SMAD molecules are latent transcription factors that interact with a plethora of different co-activators, co-repressors and polymerase. One of the well-studied cofactors that binds to nuclear SMAD trimers is SnoN. Recent data suggests that SnoN modulates the effect of TGF beta [23]. In Fig. 16 we compare the SnoN and the multi-protein complex SnoN-SMAD-tetramer concentration from the ODE with the PDE model in the cell geometry with extensions. Due to the cytosolic trimer diffusion, there are less trimers available in the nucleus than for the ODE model. So, in the nucleus more SnoN molecules and less complexes than in the ODE model can develop in time. The deviation in the complex concentration is present only from 15 to 50 min after activation which implies that due to nuclear accumulation there are enough trimer molecules available in the nucleus for complex formation when enough time is provided. The amount of the concentration deviation seems small: with the values of 0.1 nM (2 % of the total concentration) for SnoN and 0.05 nM (6 % of the total concentration) for the complex SnoN-SMAD-tetramer. Nevertheless, due to the feedback loops even small changes in nuclear SMAD trimers can have a profound effect on the multi-factor complexes. SnoN interacts also with the transcription factor p53 [24]. In conclusion the temporal availability of SMAD-trimers due to the modulatory effect of co-activators and co-repressors can result in altered cell fate in cells with irregular shape.

#### Result 15.

For cells with irregular shape the effect of diffusion is greater. The cell geometry has greater influence on the change in the nuclear concentration than the diffusion velocity.

### Inter-cellular diffusion

In contrast to the intra-cellular signaling pathways discussed above, we will see that for the inter-cellular IL-2 signaling pathway high gradients are crucial for the effective signal.

Upon stimulation by antigen in the lymph node, the proliferation and the differentiation of T lymphocytes are tightly regulated by the interplay of regulatory T cells and T helper cells. These T helper cells recognize an antigen of an antigen presenting cell and then activate responding T cells as a part of the immune response. Regulatory T cells instead suppress the activation of the responding T cells. Understanding the role of regulatory T cells is important for the treatment of autoimmune diseases and cancer. The success of organ transplantation and cancer immunotherapy are directly linked to the suppressing activity of regulatory T cells. The experimental, theoretical [14] and numerical studies [17] have identified secretion and uptake of IL-2 as a possible mechanism mediating immune suppression by regulatory T cells.

In [25] it was found that the cytokines are secreted in a polarized way at the immunological synapse preferred in a very narrow space nearby the contact with the antigen presenting cell. In particular, it is not understood within which spatial range cytokines can signal. It was speculated that the range of influence of the cytokine remains restricted to these two contacting cells. On the other side, it was shown that all cells in the lymph node can sense the cytokine IL-4 [26]. This gives the evidence to a more global distribution of cytokines.

Cytokine concentrations measured by ELISA studies in T cell cultures are typically in the pM range [27]. In this regime, cytokine molecules can reach their targets only after the diffusion over large distance as seen from the molecular scale. We assume that the time for IL-2 molecules to diffuse towards a T cell is short. In contrast, the average time to reach a receptor at the cell surface is long because naive T cells express only small number of cytokine receptors, which is not detectable in experiments. Thus, IL-2 concentrations measured by ELISA are too low to generate reliable signals. It has been reported that IL-2 is subject to huge gradients with much higher concentration at the surface of T cells [28]. To investigate such inhomogeneities, it is necessary to consider the spatiotemporal dynamics of cytokine signals. More details are given in [13].

We determine the number of activated cells (Fig. 12), the amount of IL-2 in the extra-cellular space (Fig. 13), the range of action of IL-2 (Fig. 14) and the required number of regulatory cells to downregulate the signal (Fig. 12). In our calculation we observe a variety of IL-2 amount over three orders of magnitude over the entire simulation time (Fig. 13 left). Thus, for activation the cells require both a transient strong and a stable weak IL-2 signal. The competition for IL-2 in the lymph node among regulatory T cells, responding T cells and T helper cells is thus a very local process. The position of the secretory cells decides which cell will be activated in the absence of strong stimulation. Figure 12(e), (f) show that for the chosen set of parameters 25 % of regulatory cells are enough to cancel out the high levels of IL-2 as no activated cells (marked with gold) are found.

We observed that for a large secreting rate the surrounding cells are activated as well for the spatial range over which the IL-2 signal can occur.

#### Result 16.

Secreting cells produce short-range signals despite of fast diffusion. For the chosen set of parameters, the radius of activation covers 2 cell distances (20 *μ*m). For the higher secretion rate in the Result 12 it covers 4 cell distances (50 *μ*m). Only clusters of secreting cells will cause long-range signals [13].

## Conclusion

In this work we presented the simulation results of new mathematical models for signaling pathways. Including the aspect of the diffusion of the molecules and the benefit of measured data, our models show a special structure described by a system of coupled PDE/ODE with Robin type boundary conditions. The three dimensional simulations in several realistic cell geometries with specific parameters show the dominance of the chemical reactions over diffusion in all models. The chemical reactions determine the gradient formation and their result to the cell fate and finally, the spatial range of the signal. Nevertheless, the diffusion must be considered in cells with irregular form or for slow diffusing molecules (*D*<1 *μm*^2^/*s*).
